# Computationally intelligent real-time security surveillance system in the education sector using deep learning

**DOI:** 10.1371/journal.pone.0301908

**Published:** 2024-07-11

**Authors:** Muhammad Mobeen Abid, Toqeer Mahmood, Rahan Ashraf, C. M. Nadeem Faisal, Haseeb Ahmad, Awais Amir Niaz

**Affiliations:** Department of Computer Science, National Textile University, Faisalabad, Pakistan; Mirpur University of Science and Technology, PAKISTAN

## Abstract

Real-time security surveillance and identity matching using face detection and recognition are central research areas within computer vision. The classical facial detection techniques include Haar-like, MTCNN, AdaBoost, and others. These techniques employ template matching and geometric facial features for detecting faces, striving for a balance between detection time and accuracy. To address this issue, the current research presents an enhanced FaceNet network. The RetinaFace is employed to perform expeditious face detection and alignment. Subsequently, FaceNet, with an improved loss function is used to achieve face verification and recognition with high accuracy. The presented work involves a comparative evaluation of the proposed network framework against both traditional and deep learning techniques in terms of face detection and recognition performance. The experimental findings demonstrate that an enhanced FaceNet can successfully meet the real-time facial recognition requirements, and the accuracy of face recognition is 99.86% which fulfills the actual requirement. Consequently, the proposed solution holds significant potential for applications in face detection and recognition within the education sector for real-time security surveillance.

## 1. Introduction

In recent years, facial detection and recognition have become a research hotspot in the field of computer vision [1–3]. They gain more and more attention with the continued development of AI (artificial intelligence). Meanwhile, security protocols and surveillance apps are becoming more accurate and functional because of the advancements in machine learning (ML) and artificial intelligence (AI) [4, 5]. It is frequently used for matching identities and video surveillance. Standard facial detection and identification techniques have been utilized in different public sectors, including access control systems, criminal investigation systems, education sectors, and more. In the education sector, real-time security surveillance and facial recognition are crucial for enhancing campus safety by swiftly identifying and responding to potential threats. The implementation of real-time security surveillance and facial recognition contributes to robust access control systems, detecting unauthorized persons and ensuring the safety of students, faculty, and staff.

Moreover, security surveillance can assist in the rapid identification of individuals in the university area, facilitating timely responses and improving overall situational awareness. Due to a heightened sense of security, there has been an increase in video surveillance technologies in private and public areas in recent years, such as RFID and CCTV [6]. Facial detection and recognition, as well as retina comparison and fingerprint, are all part of the biometric system. The use of multiple parameters for authentication during surveillance is a hot topic in the field of security research [7–10]. Facial recognition for authentication is one of the most important biometric verification factors. In many public and private sectors, real-time manual observations are still used to check areas for possible suspects. The manual labor involved, as well as the potential for human error, make the security system less efficient. Regarding this matter, organizations face a number of challenges. Traditional video surveillance systems are primarily built for offline analysis of recorded video streams and rely heavily on human operators for processing. There are various flaws in video surveillance systems, including unclear images, poor stability, complex structures, a large amount of storage space required to retain surveillance data, and relatively high prices. These systems are only utilized on a small scale as biometric [11–14]. The problem with real-time monitoring is that an event can easily pass unreported due to false or simultaneous alerts and a lack of time to playback and evaluate all potentially important video feeds [15–18]. The performance of the system is also affected by environmental changes like weather and lightning. False alarms are another problem. Authorized individuals can also be denied in the event of a false alarm. Video surveillance systems have a number of flaws, including complex structure, poor stability, blurry images, and require a lot of storage for surveillance data. Current systems are both complex and costly. The traditional algorithms of facial detection and recognition include MTCNN [19], AdaBoost [20], Haar-like [21], etc. For face detection, these algorithms use facial geometry features and template matching, which is challenging in terms of detection speed and accuracy.

This study proposes a facial detection and recognition system based on RetinaFace [22] and FaceNet [23] to address the above-stated problems. RetinaFace is a more advanced face detection algorithm that provides precise bounding box localization. RetinaFace is computationally intelligent due to its ability to detect faces with high accuracy, even in challenging conditions such as occlusions, different poses, and varying lighting conditions [22, 24]. RetinaFace achieves this using a multi-task learning approach, where it simultaneously predicts facial landmarks, face attributes, and face-bounding boxes. The multi-tasking ability of RetinaFace captures rich facial information, making it more computationally intelligent in understanding the complex features of a face. FaceNET uses the triplet loss function during the training phase, which takes into account the relative similarity between three images: an anchor image (a specific face), a positive image (another instance of the same person), and a negative image (a different person) [23]. The triplet loss function helps the model learn a more discriminative feature space where similar faces are clustered together, and dissimilar faces are pushed apart. Hence, the FaceNET approach significantly improves the accuracy of face recognition, making it more computationally intelligent in distinguishing between similar faces.

This research aims to propose an algorithmic configuration demonstrating computational intelligence through innovative methodologies in face recognition tasks using RetinaFace and FaceNet. In the proposed study, RetinaFace is combined with FaceNet to create a comprehensive face-processing pipeline that addresses both face detection and recognition. The baseline FaceNet model focuses solely on face recognition, generating embedding to facilitate accurate matching. The integrated RetinaFace and FaceNet model incorporates RetinaFace for face detection, providing precise bounding box localization of faces in digital images. This integrated approach enhanced the overall system’s versatility, enabling it to handle diverse facial poses, scales, and occlusions during the process of detection, followed by accurate recognition using FaceNet. The baseline FaceNet model, being a foundational face recognition model, lacks the specific face detection capabilities that RetinaFace contributes to the integrated model. Therefore, the combined RetinaFace with FaceNet model offers a more comprehensive solution for applications requiring both face detection and recognition functionalities. The proposed technique based on RatinaFace and FaceNet offers a comprehensive security framework that enables continuous monitoring of designated premises. It is designed to promptly notify the security team of any anomalous activity, such as the presence of suspicious individuals or items within a given area. Additionally, the proposed technique reduces false alarms and improves data management and information extraction from large amounts of surveillance data instead of large video recordings. The prominence of the proposed face detection and recognition technique against several existing methods is: a) the efficient detection and recognition of faces with different poses; b) the detection and recognition of faces with different occlusion rates such as 20%, 40%, 60%, and 80%; c) robustness in the detection and recognition of multiple faces in a single image.

This paper is organized as follows: In Part 2, a brief overview of existing work in the area of deep learning for detecting and recognizing face images is provided. Part 3 outlines the proposed methodology for face detection and recognition. Part 4 provides a brief discussion on training, testing, and experimental results. Finally, Part 5 presents the conclusion.

## 2. Literature review

Due to the enormous increase in everyday security threats and problems, investigating the contents of surveillance videos has become a significant research field. Identification and recognition of human faces for authorization is one of the most effective ways to mitigate security threats and problems. In literature, a semi-automated Viola-Jones algorithm was proposed for face recognition [25]. Over the years, improvements in this algorithm were proposed using the wavelet-based function to improve real-time face recognition [26, 27]. In the frameworks [26, 27], the first stage is the detection and location of the movement. The remaining stages rely heavily on the detection and recognition of faces. The moving human face in surveillance videos is detected and localized using the background subtraction models [28]. Background subtraction models have several limitations consisting of signal-to-noise ratio due to low-resolution cameras, blurring effects resulting from jittering movement in the camera optics, noise found in the surrounding environment, compression, adjustment of illumination, and others [29].

The Gaussian mixture version can also be used for background subtraction [30]. Multimodal mean real-time video stream background modeling approaches are also contributing to the developments in image processing and machine learning [31]. Gaussian mixture model (GMM), in combination with background modeling approaches, has been proposed to handle changes that take place by minute movements. Other segmentation techniques, such as time-based changing models, utilize pixel differences between two to three frames to extract successive shifting regions. In [32], a dynamic background modeling approach utilizing a neuro-fuzzy algorithm and adopting the concept of a time-based changing model is proposed to reduce shadows.

Feedforward algorithms mostly depend on skin color data for face recognition [33]. Skin color pixels have values between 0.36 < *r* < 0.456 and 0.28 < *g* < 0.363 [32]. For skin color pixel recognition, the Gaussian and histogram models are used to overcome singular limitations and improve accuracy by up to 91%. The hue, saturation, and value (HSV) color model is used to represent pixel classification, with a range of H from 1 to 51 and a range of S from 0.21 to 0.69 [34, 35]. The YCbCr and HSV models are fused. Accordingly, the range of *Cb* ≤ 125 or *Cb* ≥ 160 and the range of *Cr* ≤ 100 or *Cr* ≥ 135. The *H* ranges from 26 to 220. The skin-shading approach is sensitive to luminance variations. This technique shows ineffectiveness if the background comprises skin shading-like objects. To identify the pixels of skin color the facial recognition generates the face edge map and skin-shading threshold value through the YCbCr model and Viola-Jones approach to validate the recognition. Viola-Jones organized the rectangular face highlights using a constant Adaboost calculation [26, 36]. Progressive HEER characteristics are used to adjust the diversity. To reduce the number of false positives, skin color depth information is combined with the Viola-Jones face identification. A person’s personality is verified through a request to match with a face coordinate in the database during the acknowledgment step, which comes after facial identification.

As facial recognition is important in many fields, therefore diverse approaches are required to accomplish this task. Thus, neural networks (NN) that work in parallel and simple components to perform different operations. Gender classification, human facial emotion, and many other tasks can also be accomplished using neural networks. The NNs work well on the images with varying lightning effects and offer improved accuracy. The main disadvantage of the neural network (NN) is the amount of time required for training. ANN [37, 38] recognizes the face by learning from prior experience. Face recognition is also accomplished by combining NN and progressive intelligence [39, 40]. The probabilistic neural network (PNN) [41] technique recognizes after detecting the faces from grayscale images containing front faces. The most significant benefit of using PNNs is that they require minimal time for training. However, this technique deals only with the frontal faces. A self-organizing map neural network (SOM) [42] is an artificial neural network that has the feature of topological preservation and is used in facial recognition. Following the feature engineering stage from facial images, the FFNN and RBFN are common NN classifiers that make the facial recognition process easier and more precise. However, both the FFNN and RBFN are computationally expensive [43, 44].

After a thorough analysis of the literature, various challenges can be summarized as follows: First, the protection checks on authorized individuals are quite uncomfortable. Secondly, frequent changes in authentication methods may affect the use of normal systems and, in general, may be hazardous. Thirdly, finding an unauthorized person might be quite difficult, and the proposed technique can be helpful in this process. Lastly, a wrong alarm timing may be a common issue [45]. Thus, to achieve better results, the proposed technique may be an acceptable solution to the current shortcomings.

## 3. Proposed method

**Fig 1:**
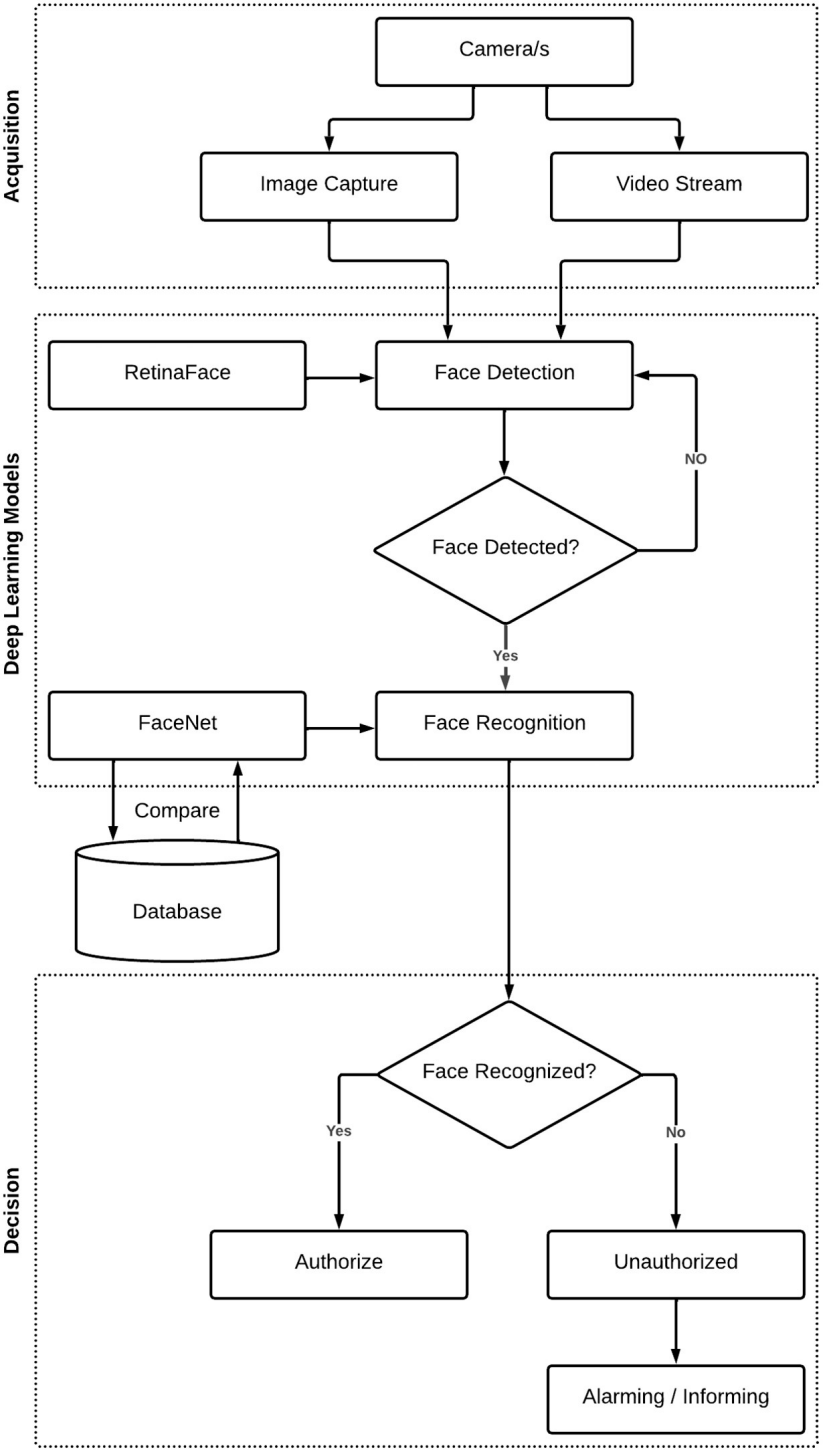
Proposed system for real-time security surveillance.

Various methods have been discussed in section 2. The drawbacks in the studies and different approaches for solving these drawbacks are described subsequently. The main elements of the devised methodology are shown in Fig 1.

Fig 1 demonstrates the model’s usage of video data obtained by a high-definition CCTV camera. After that, image acquisition generates the image’s keyframe. Then, facial detection occurs when the image’s foreground and background are extracted. Moreover, the detected human face from the image must be localized. Following face localization, face recognition compares existing images in the database. The current research is useful in this context. The proposed method is applied to reduce false alarms and improve data management and information extraction from large amounts of surveillance data. Finally, the extracted image will be compared to the images previously stored in the database. In the absence of a match, a security signal or alarm is triggered for further action, and the system starts recording a particular face that is not present in the database. The database is updated with the currently processed image parameters. The stored facial data may be utilized in the future to maximize source location identification, and time consumption, and reduce complexity.

### 3.1 Design and create a database

**Fig 2:**
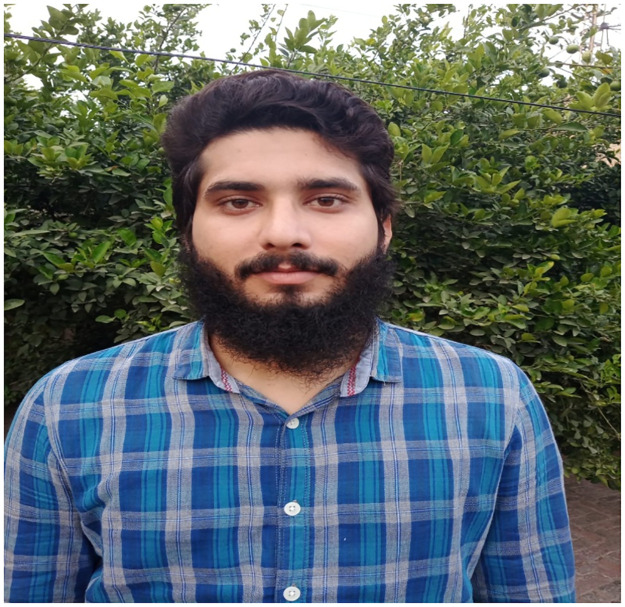
Using a simple image as the input.

The proposed system utilizes CCTV cameras in a real-time environment for image acquisition. The face recognition algorithm is based on real-time streaming from the digital camera. The highlighted process extracts a single frame from real-time streaming. This image acquisition window size is 480 x 640 pixels. The first and most important part of the face recognition task is human face detection. Locating every human face in an image is the primary goal of human face detection. During the pre-processing phase, the system determines whether a face is present in the resulting image. The human face is verified repeatedly from the input data by the system. If a face cannot be identified in the keyframes of real-time streaming, the system will automatically repeat previous commands until a human face is identified (Fig 2).

**Fig 3:**
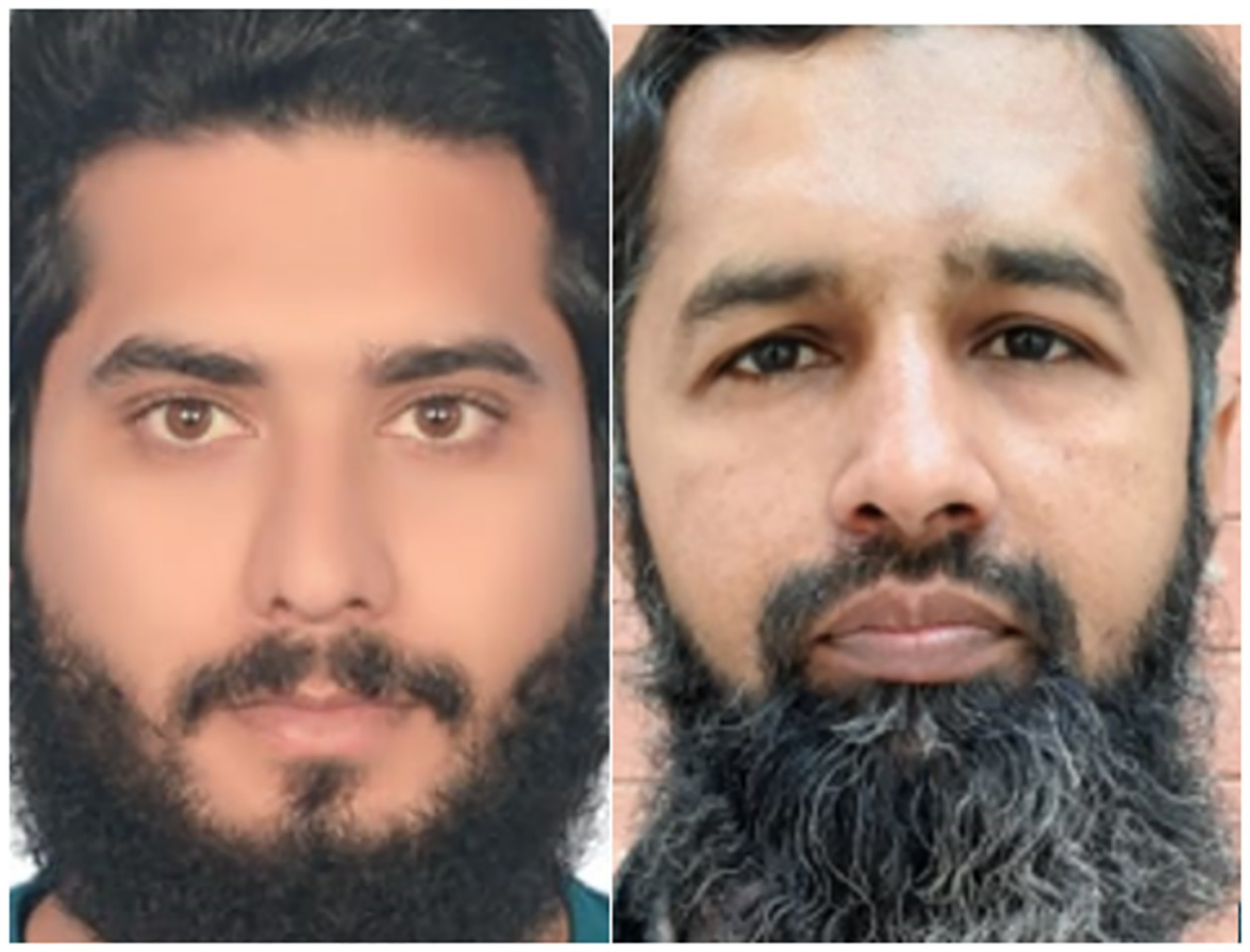
Extracted face from human image.

In the next step, the human face is extracted from the keyframe, as shown in Fig 3. The facial image cropped from the original keyframe is just a small part of a human face. The database is created with the facial images. Two separate datasets are used in the system when working offline. These systems consist of the AT&T datasets [46, 47] and Chokepoint Faces [32] systems.

The camera is made to provide a live video feed when it is directly connected to a network or computer. A camera typically lacks its storage memory; therefore, it depends on the available memory storage to function. We utilized a standard HP Wide Vision HD camera for this study. The specs are given for the cameras utilized in the execution of the research.

Face detection in the images was done using the camera. The system proceeds by acquiring images. Then, the object detector captures the image cascade using the Viola-Jones method to identify various facial features, such as the mouth, nose, eyes, etc. In the developed system, image labeling can also be used to train a neural network (NN) classifier. The identified face feature is subsequently saved in a database along with the name of an authorized individual and a face image. A real-time system detects each facial image after a 2-second delay.

### 3.2 Pre-processing

The actual size of the captured image frames from the video using CCTV cameras was 1280 × 1020 pixels. Image frames with such a large resolution size are computationally expensive. Therefore, the image frames were resized to 350 × 350 dimensions.

### 3.3 Facial detection and localization

**Fig 4:**
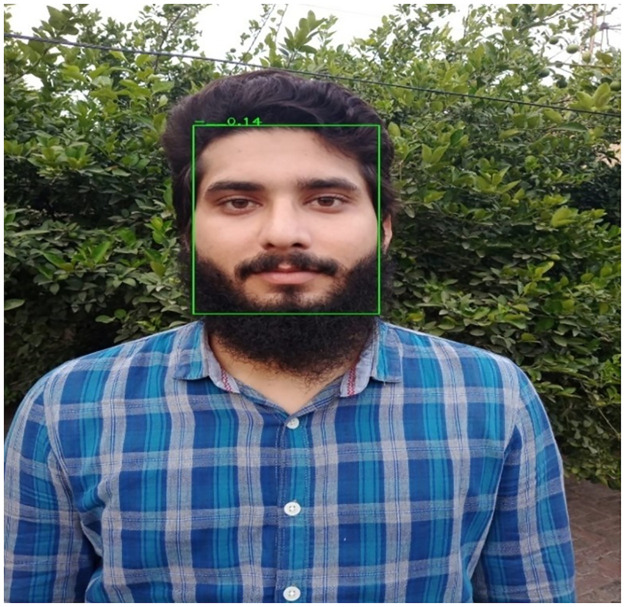
Process of face detection.

Facial detection is the first and most important part to find if there is any human face in an image or not [48]. Several types of techniques are available for this purpose. These techniques essentially identify different face features, including the mouth, iris, nose, nostrils, eyes, and eyebrows. The techniques identify these features before identifying the face region [49]. This process of face detection is shown in Fig 4. This research proposed RetinaFace is an approach designed to solve both face identification and face alignment problems. This method is a single-shot framework that does pixel-wise face localization by implementing three subtasks such as facial detection, 2D facial alignment, and 3D face reconstruction utilizing a mesh decoder.

RetinaFace consists of three main phases: feature pyramid network, context module, and multi-task loss. The Feature Pyramid Network handles features at different scales, a context module incorporates contextual information, and a multi-task loss function trains the model to perform multiple tasks simultaneously. The working of these components is explained in the following subsections.

**Fig 5:**
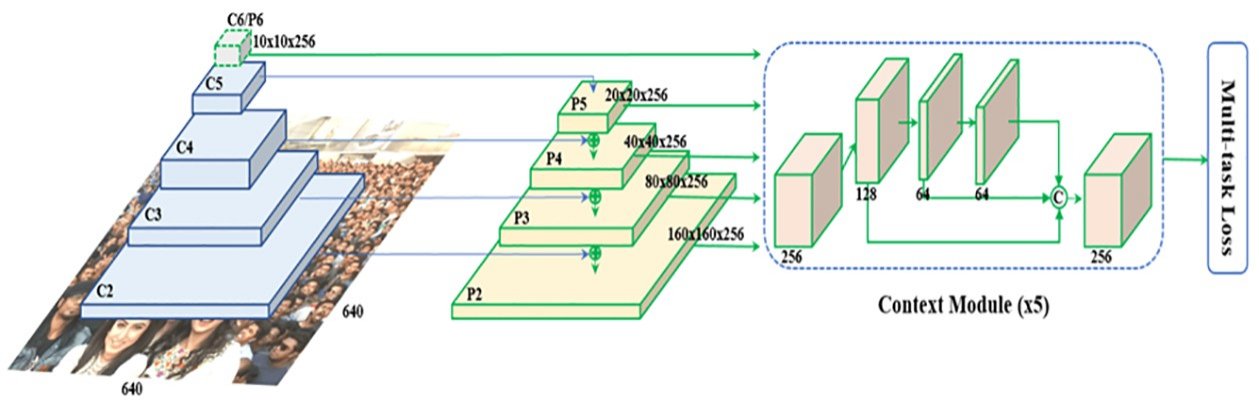
Architecture of RetinaFace.

RetinaFace is a deep learning-based technique that can detect the human face in a facial image by performing multiple facial localization tasks. Facial box prediction, 2D face landmark localization, and 3D facial reconstruction are all performed by RetinaFace techniques under a single target [22]. Fig 5 shows the complete architecture of the RetinaFace technique [22].

The RetinaFace method consists of three phases:

1. Feature Pyramid Network,
2. Context Module, and
3. Multi-task Loss Function.

#### 3.3.1 Feature pyramid network

It uses a single facial image as an input and generates outputs of five feature maps at various scales. It defines the feature pyramid levels, ranging from P2 to P6. These levels are computed utilizing the top-down and lateral connections in the output of the ResNet residual stage, i.e., C2 through C5. It includes applying 3×3 convolution with a stride of 2 to the appropriate ResNet residual stage [22].

#### 3.3.2 Context module

To improve the rigid context modeling power and expand the receptive field, the context modules are built separately on each of the five feature pyramid levels. The DCN (Deformable Convolution Network) is used in place of the 3×3 convolution layers within the context modules and lateral connections. The DCN enhances the flexibility of non-rigid context modeling [22].

#### 3.3.3 Multi-task loss

The efficiency of the facial localization process is increased by using cascade regression with multi-task loss. Initially, the context module determines the bounding box using regular anchors. The second one uses the regressed anchors to estimate a bounding box that is more precise [22]. The overall multi-task loss is calculated as follows:

$$L=\;L_{cls}\left(a_{j},\;a_{j}^{*}\right){+}_{1}a_{j}^{*}L_{box}\left(b_{j},\;b_{j}^{*}\right)\;{+}_{2}a_{j}^{*}L_{pts}\left(m_{j},\;m_{j}^{*}\right)\;{+}_{3}a_{j}^{*}L_{pixel}$$ (1)

**Fig 6:**
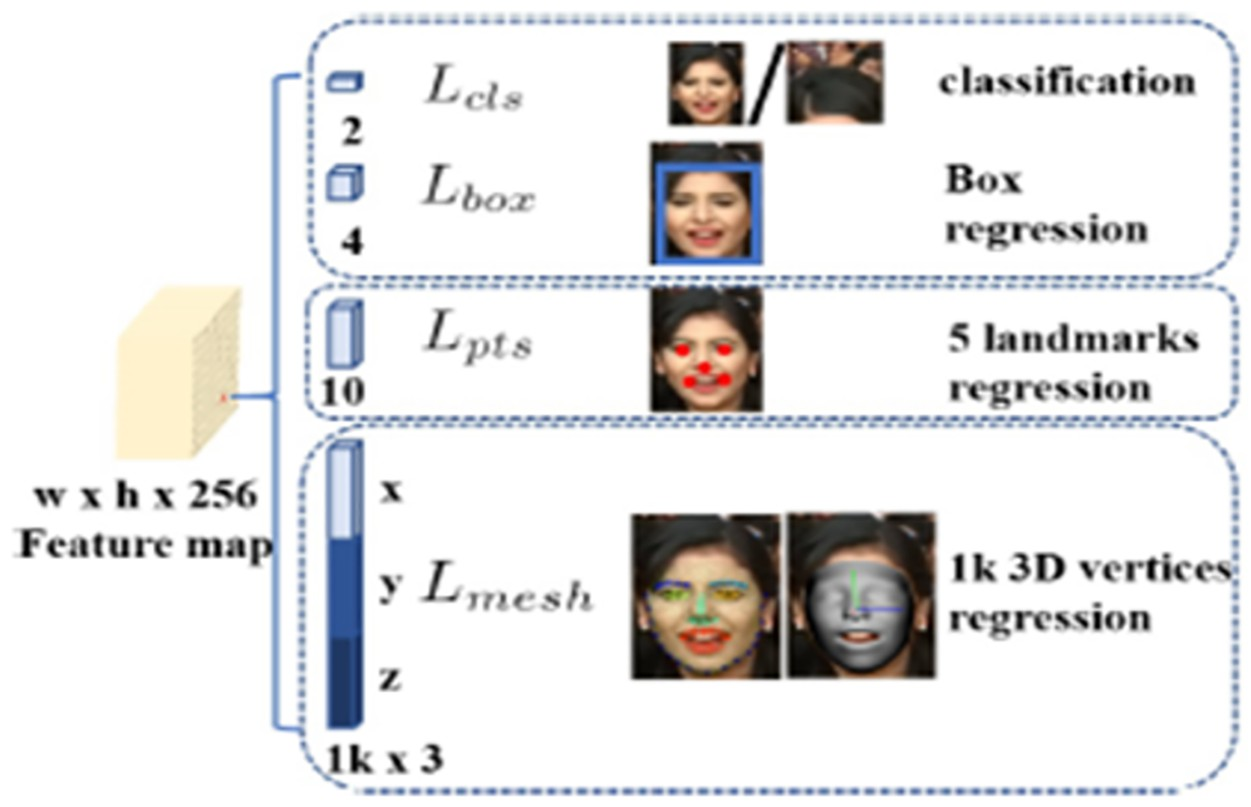
RetinaFace multi-task loss function.

Here, the SoftMax loss is represented as $L_{cls}$ as a binary class (face or not face). The facial classification loss is represented as $\left(a_{j},\;a_{j}^{*}\right)$. *a*_*j*_ is a predicted probability of anchor j will be a face. $a_{j}^{*}$ is 0 for a negative anchor and 1 for a positive anchor. The loss for the coordinates is represented as $L_{box}$ in every predicted boundary box. The facial box regression box is represented as $\left(b_{j},\;b_{j}^{*}\right)$. The predicted box’s coordinates are related to the position anchor, which represents *b*_*j*_. The ground-truth box’s coordinates are related to the position anchor, which represents $b_{j}^{*}$. The loss for the coordinates is represented as $L_{pts}$ in the facial landmark. The facial landmark regression loss is represented as $\left(m_{j},\;m_{j}^{*}\right)$. The predicted five facial landmarks represent *m*_*j*_. The ground-truth is related to the position anchor, which represents $m_{j}^{*}$. The dense regression loss is represented as $L_{pixel}$. The RetinaFace loss function is shown in Fig 6 [22].

### 3.4 Facial recognition

For problems including face recognition, verification, and clustering, FaceNet offers a unified embedding phenomenon. In other words, a picture of person A will be placed closer to all the other photographs of person A as compared to images of any other person available in the dataset. It maps each face image into an Euclidean space so that the distances in that space correspond to face similarity.

FaceNet differs from other systems primarily in that it learns the mapping from the photos and generates embeddings without utilizing any bottleneck layers for tasks like recognition or verification. Once the embeddings are made, they can be used as the feature vector for all subsequent activities, including verification and recognition, which can be completed using industry-standard methods. For instance, by using embeddings as the feature vector, we may utilize k-NN for face recognition. Similarly, we can use any clustering algorithm to group the faces, and all that is required for validation is the definition of a threshold value.

**Fig 7:**
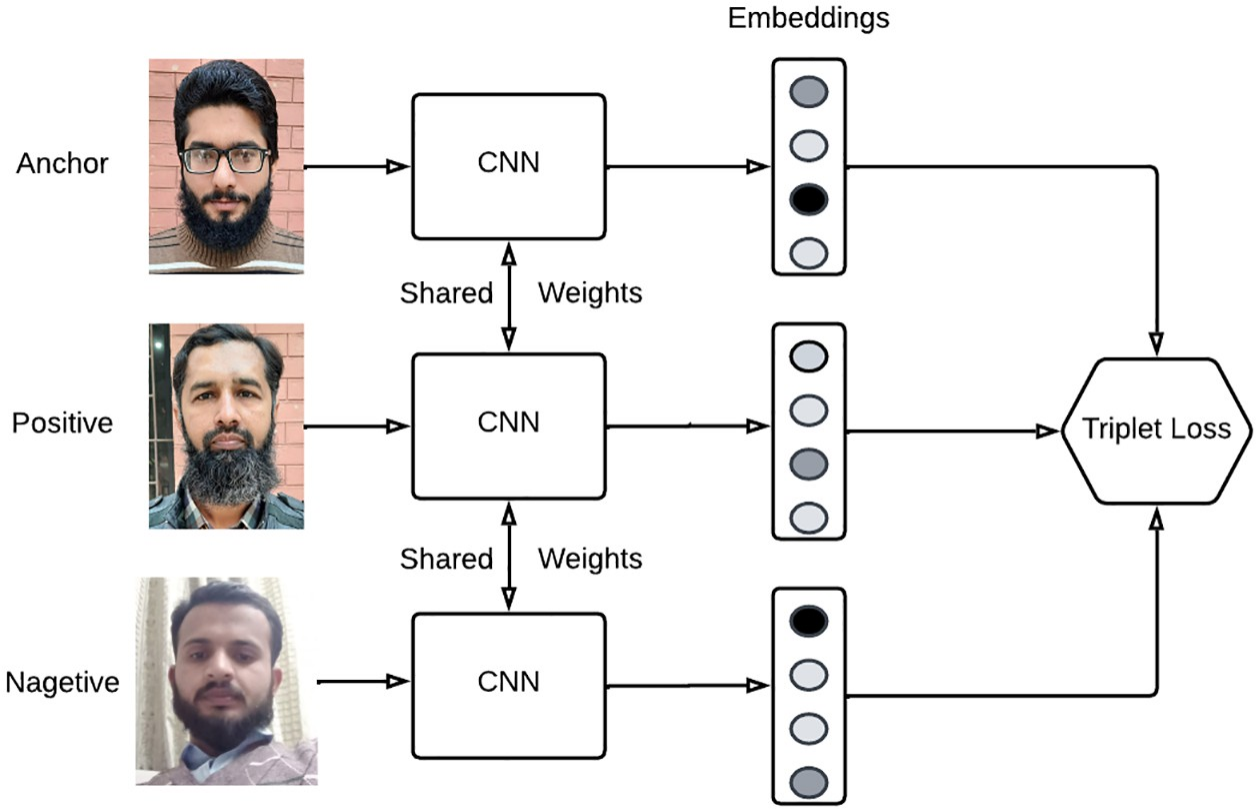
Triple loss function.

FaceNet uses deep convolutional neural networks (CNN). The network is trained so that face similarity corresponds to the squared L2 distance between the embeddings. The photographs that are utilized for training are resized, altered, and have the face region severely trimmed. The loss function of FaceNet is another crucial component. It takes advantage of the triplet loss function (see Fig 7). Three images anchor, positive, and negative, are required to calculate the triplet loss. In the part after this one, we will go into great detail about triplet loss.

#### 3.4.1 Triplet loss and selection

**Fig 8:**
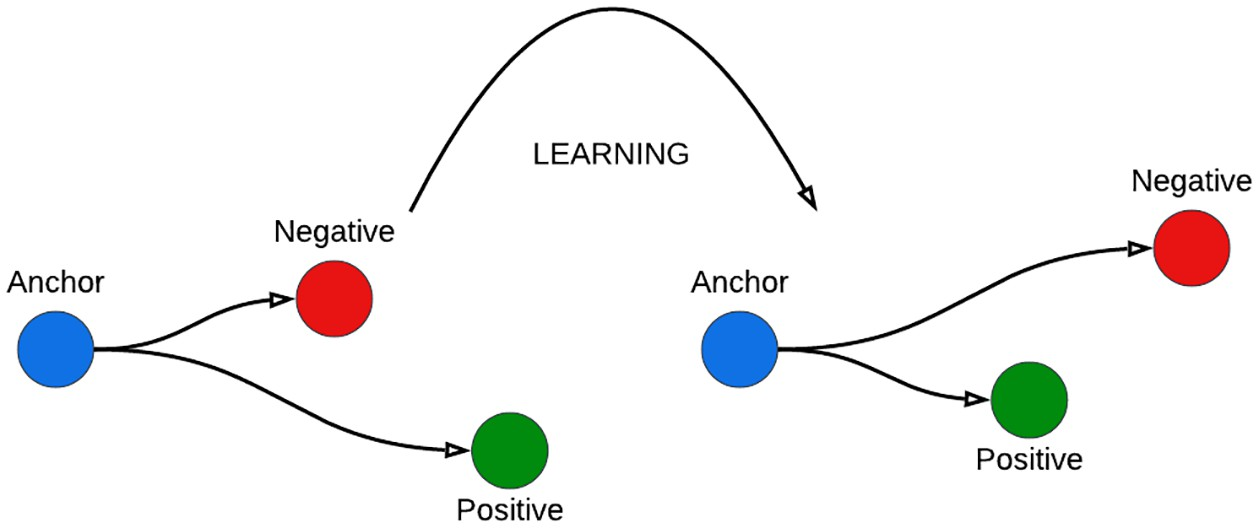
Triple loss and selection.

The idea behind the triplet loss function is that we want our anchor image, which is an image of a particular individual named A, to be closer to positive images than to negative ones (Fig 8).

In other words, we would like the distances between our anchor picture and the embeddings of our positive images to be smaller than those between our anchor image and the embeddings of our negative images.

Formally, the triplet loss function is described as:

$$\overset{N}{\underset{i}{\sum }}\left[{\left\|f\left(x_{i}^{b}\right)-f\left(x_{i}^{m}\right)\right\|}_{2}^{2}-{\left\|f\left(x_{i}^{b}\right)-f\left(x_{i}^{l}\right)\right\|}_{2}^{2}+\alpha \right]$$ (2)

Where $f\left(x_{i}^{b}\right)$ represents the anchor image, $f\left(x_{i}^{m}\right)$ represents positive image, and $f\left(x_{i}^{l}\right)$ represents negative image. α represents the margin between positive and negative pairs before and after training. In Eq 2, anchor, positive, and negative pictures are shown by the superscripts *b*, *m*, and *l*, respectively.

The margin between positive and negative pairing depends on how the alpha is defined in this context. A threshold value fundamentally determines the difference between our image pairings. The difference between our anchor-positive and anchor-negative image pairings should be at least 0.5 if, for example, alpha is set to 0.5.

#### 3.4.2 Triplet selection

The selection of the appropriate image pairs is crucial since there will be many image pairs that match this criterion, which means that our model will not learn much from them and will also lead it to converge slowly. It is essential to choose triplets that defy the triplet limitation to guarantee rapid convergence.


$$argmax\;\overset{N}{\underset{i}{\sum }}\left[{\left\|f\left(x_{i}^{b}\right)-f\left(x_{i}^{m}\right)\right\|}_{2}^{2}\right]$$ (3)

$$argmin\;\overset{N}{\underset{i}{\sum }}\left[{\left\|f\left(x_{i}^{b}\right)-f\left(x_{i}^{l}\right)\right\|}_{2}^{2}\right]$$ (4)

According to Eq (3), we should seek out a positive image of Person A such that the gap between the two images is as great as possible given the Anchor Image of Person A.

According to Eq (4), we must locate a negative picture such that the distance between it and the anchor image of Person A is as little as possible.

So, in this case, we are just choosing the hard positives and hard negatives. With this method, we can accelerate convergence as our model picks up relevant representations. However, there is a drawback to this strategy: computing hard positives and hard negatives throughout the full dataset is computationally impractical. Calculating the hard positives and negatives for a mini-batch is a creative remedy in this situation. Here, we will pick between 1000 and 2000 samples (the batch size in the majority of tests was about 1800).

## 4. Experiments and results analysis

In this section, the detailed exploration of the proposed research outcomes is described. Beginning with an overview of the experimental environment, we delve into the intricacies of the dataset employed. Subsequently, a thorough analysis of the performance of RetinaFace is presented, followed by an in-depth examination of FaceNet efficacy. This multifaceted analysis aims to provide a comprehensive understanding of the experimental setup and the final outcomes achieved by our proposed methodology.

### 4.1 Experimental environment

All the algorithms utilized in this research are trained under the Windows 10 operating system and TensorFlow-GPU deep-learning architecture. The CPU model is Nvidia GeForce RTX 2080 TI, 32GB memory, and AMD Ryzen 7 5800X 8Core-Processor graphics card. The entire code of these algorithms is in Python 3.6 using the Anaconda environment.

### 4.2 Dataset

**Fig 9:**
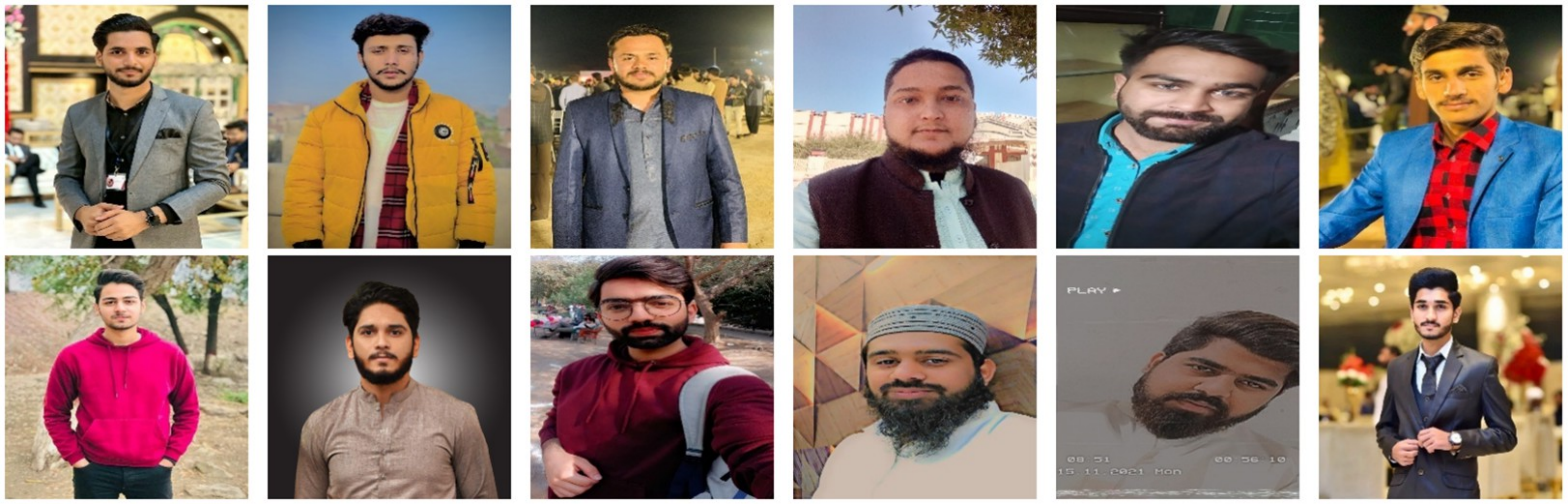
Sample image dataset for training.

To verify the effectiveness of the facial recognition algorithm discussed in this research, the IMDB-WIKI facial dataset is used to train the RetinaFace model. This dataset has 20284 human images and contains 523,051 faces with pose and occlusion marked and high variability in scale. Furthermore, the Labelled Face in the Wild (LFW) dataset is chosen as the FaceNet training set. The dataset contains 5749 people images with 13233 faces. In this dataset, 1680 people have two or more images. Moreover, this research utilized a camera to get a training image of 162 university students and staff. They contain 200 facial images, and 19 people have two or more images. The additional training sample set for detection and recognition is shown in Fig 9. All training samples go through the same pre-processing procedure.

### 4.3 Experimental analysis using deep learning models

#### 4.3.1 Analysis of RetinaFace performance

Facial detection is a very critical step before facial identification. To verify the performance of RetinaFace in facial detection, this research randomly selected 20% of the IMDB-WIKI facial dataset for validation purposes, 60% for training, and the remaining 20% for testing. Before training, some critical parameters must be initialized. The batch size is 200, and the initial learning rate is 0.1. The optimization of the network parameters is performed through the Adaptive moment estimation (Adam) [50]. It extends the stochastic gradient descent (SGD) method and is crafted to adjust the weights of a neural network throughout the training process, and the learning rate varies accordingly. The weight decay coefficient of 0.0002 is employed. The training process involved a total number of 500 epochs with a maximum of 20,000 iterations.

**Fig 10:**
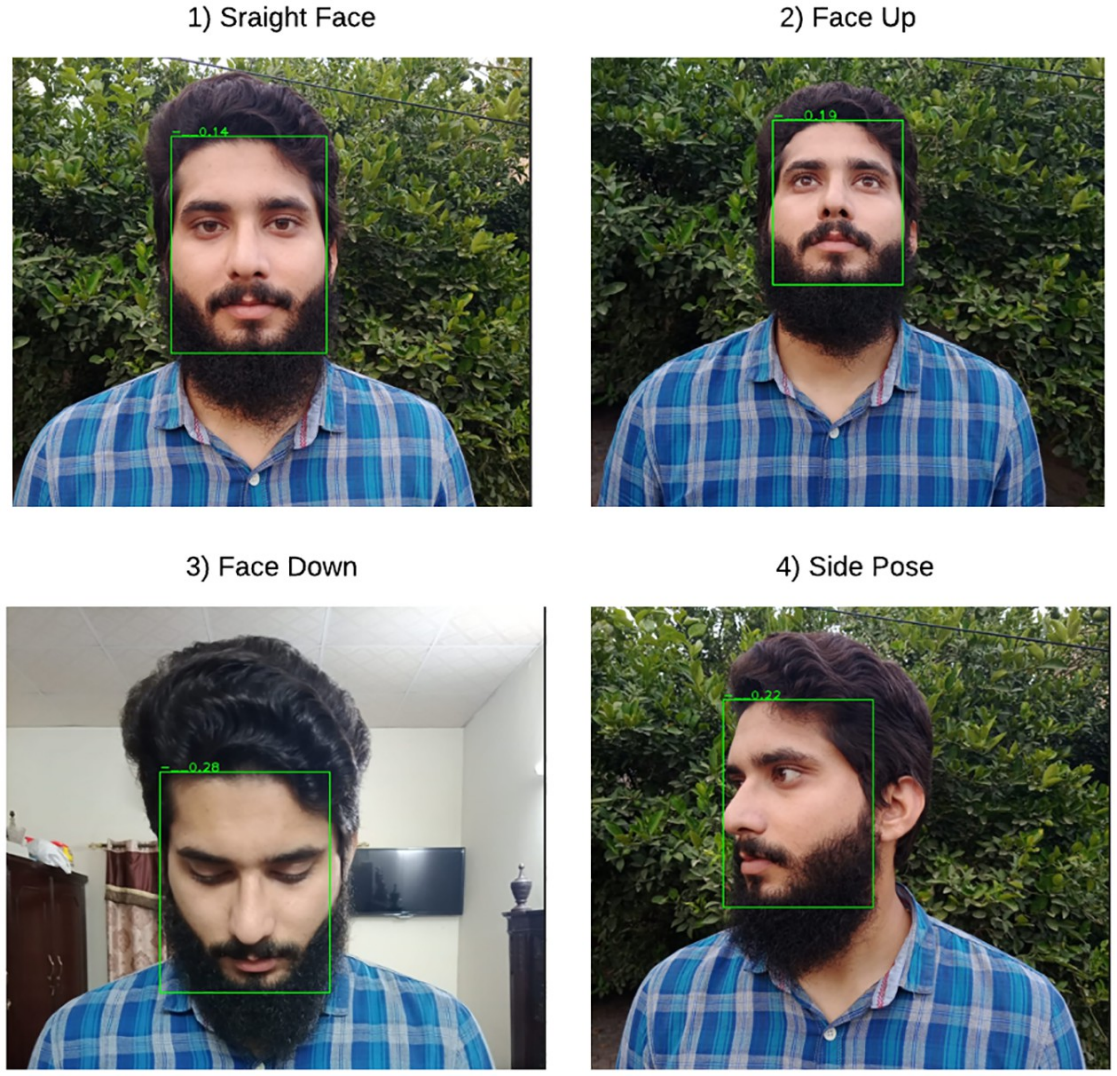
Single face detection with various poses.

This research employs data augmentation processing to improve the model robustness, such as mirror, scale variation, rotation, and so on. Fig 10 represents the detection results using RetinaFace of a single person’s face in various poses (such as straight, side pose, face up, face down). Fig 10 illustrates how RetinaFace does an accurate regression on the facial frame. Furthermore, simulation analysis compares multiple common facial recognition methods (such as Haar-like [21], AdaBoost [20], and MTCNN [19]). The simulation analysis outcomes are displayed in Table 1. RetinaFace is better than other facial detection algorithms in terms of accuracy and detection speed.

**Table 1:** Performance comparison between face detection techniques.

Method	Test samples	Test time (min)	Detection accuracy, %
Haar-like	4060	13.8375	85.76
AdaBoost	4060	9.341	88.12
MTCNN	4060	1.0625	95.37
RetinaFace	4060	0.8375	96.88

**Fig 11:**
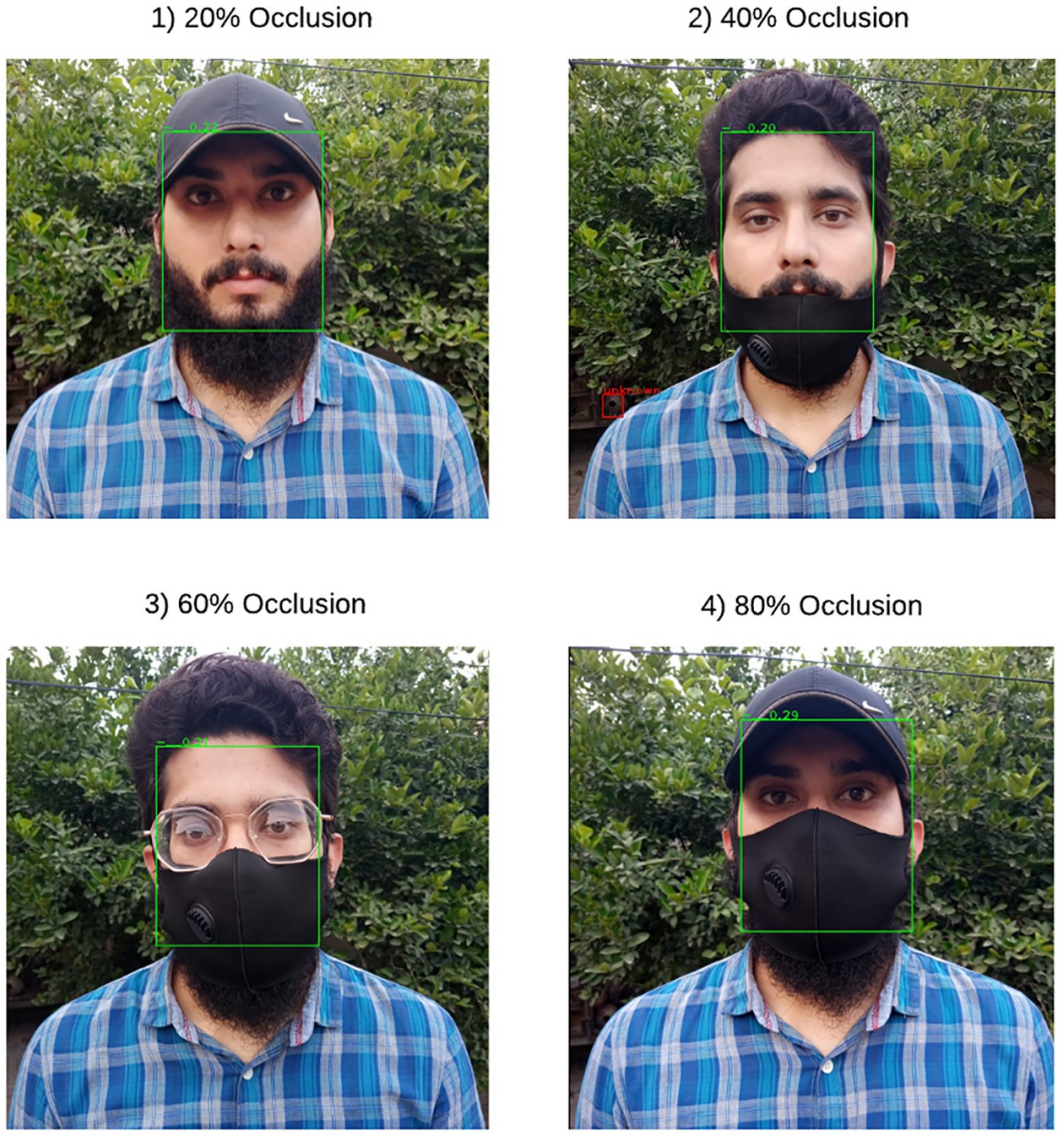
Single face detection on different occlusion rate.

Fig 11 displays the Retina Face’s detection outcomes when the rate of face obscured differs (such as 20%, 40%, 60%, or 80%). Fig 11 clearly shows that when the part of the face is obscured, RetinaFace performs very well and detects faces successfully. The experimental results of several methods for various obscure rates are displayed in Table 2. When compared to these well-known facial identification algorithms, it is clear that RetinaFace offers significant speed and accuracy improvements. Additionally, RetinaFace is lighter than other well-known algorithms, the facial detection speed is very quick, and facial alignment and facial detection are finished simultaneously. Before facial recognition, they also deliver a solid foundation.

**Table 2:** Performance comparison between different occlusion rates.

Method	Test sample	Test accuracy (80%)	Test accuracy (60%)	Test accuracy (40%)	Test accuracy (20%)
Haar-like	4060	53.87	66.45	76.31	81.34
AdaBoost	4060	60.72	73.32	83.21	88.79
MTCNN	4060	74.96	84.54	91.86	95.37
RetinaFace	4060	76.70	86.34	93.10	96.88

#### 4.3.2 Analysis of FaceNet performance

**Fig 12:**
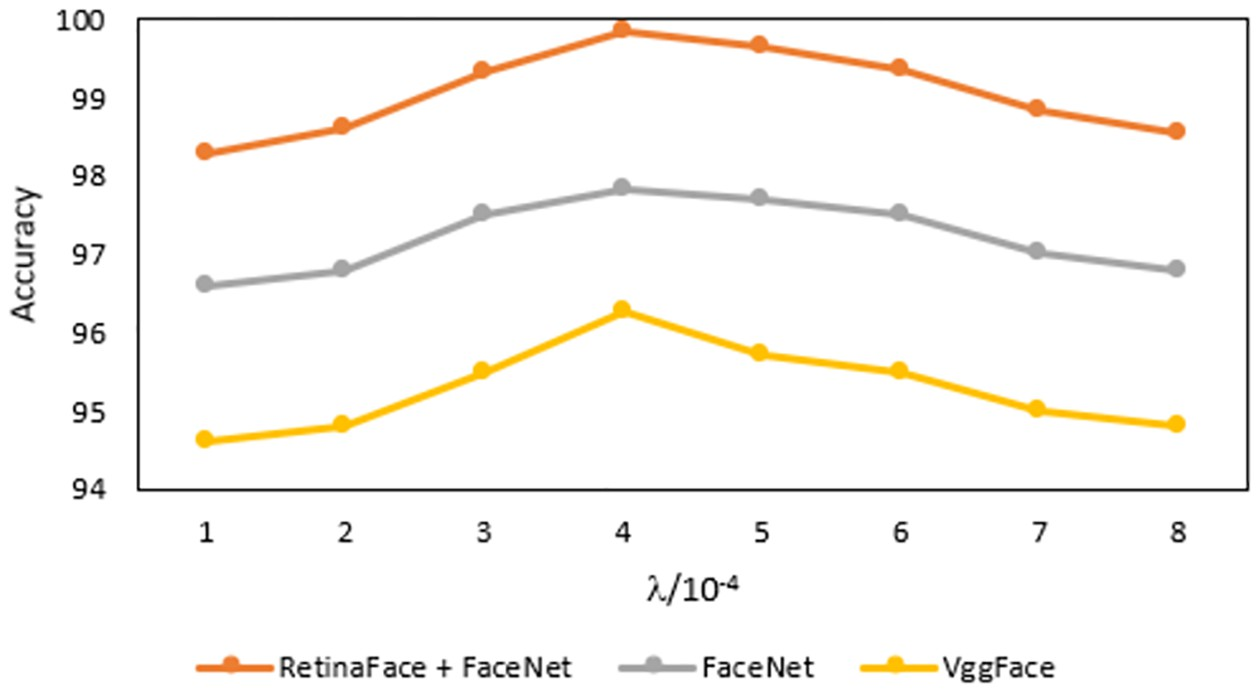
Accuracy of facial recognition techniques on different λ value.

The confirmation section is facial Recognition, FaceNet network utilized a triple loss function to train the algorithm. This training section is the same as the previous training section of the facial detection algorithm. This research randomly selected 20% of the Labelled Faces in the Wild (LFW) facial dataset for a validation purpose, 60% for training, and the remaining 20% for testing. The parameters of the models are initialized as follows: the batch size is 200, the initial learning rate is 0.05, the weight decay coefficient is 0.0005, and the maximum iterations are 30,000. By performing mirroring, scaling variation, rotation, and other operations on the LFW dataset to increase the number of training samples,

This research examines the effect of various λ values on facial recognition accuracy since varying balance coefficients will have an impact on the algorithm’s ability to recognize human faces. Fig 12 displays the comparison of facial recognition accuracy between two methods (Vggface, FaceNet, and RetinaFace fused with FaceNet) for various *λ* values on the Labelled Face in the Wild (LFW) dataset. The graphical representation shows how the facial recognition accuracy of the three methods fluctuates with the variation of *λ*. The highest facial recognition accuracy is 99.86% when λ is 3 × 10^−4^. Meanwhile, the model reaches minimum facial recognition accuracy when the *λ* equals to 0, which utilizes triplet loss. RetinaFace fused with FaceNet performs better than MTCNN fused with FaceNet algorithms, so they verify the efficacy of hybrid training.

**Fig 13:**
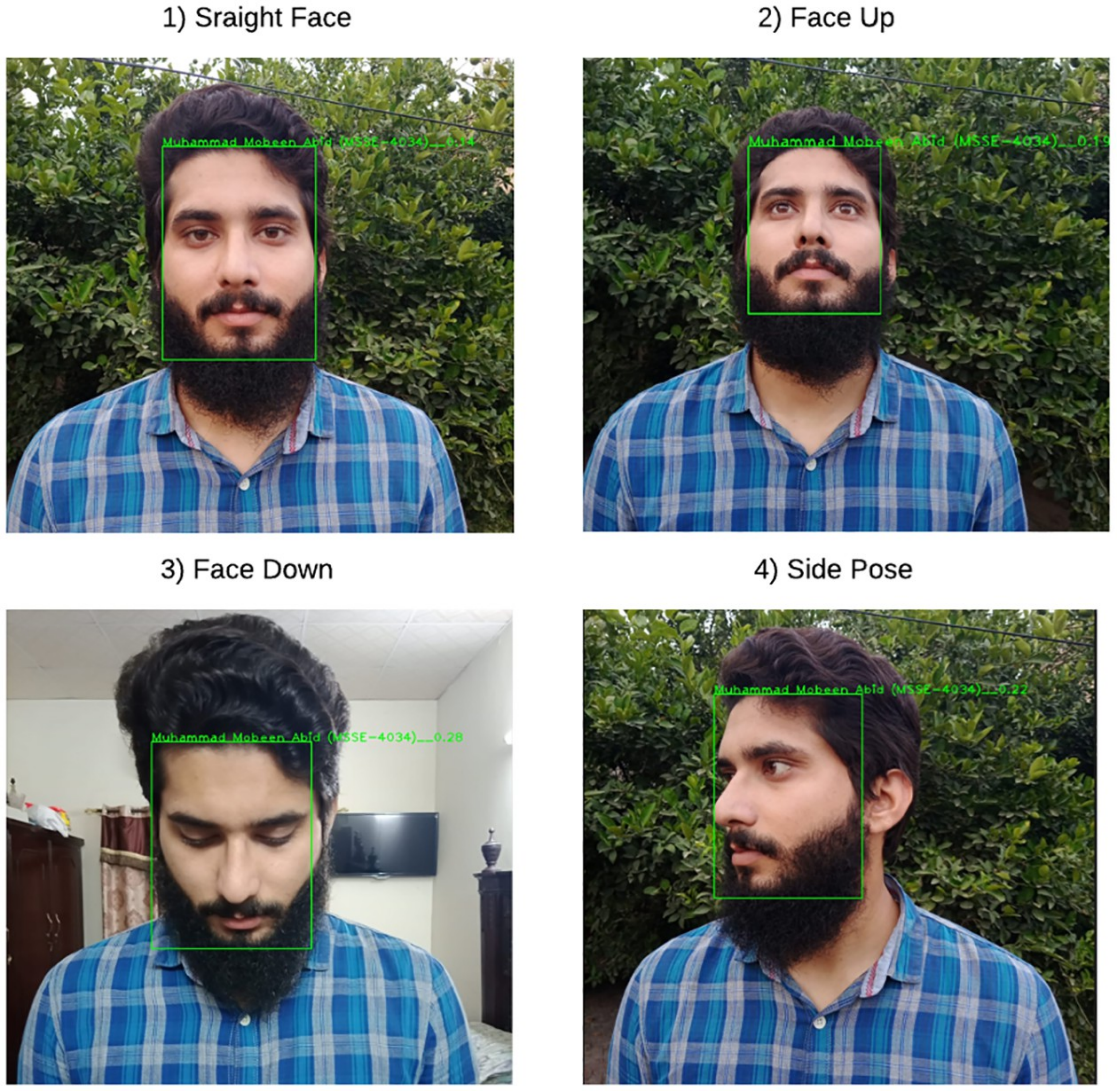
Single face recognition result with various poses.

**Fig 14:**
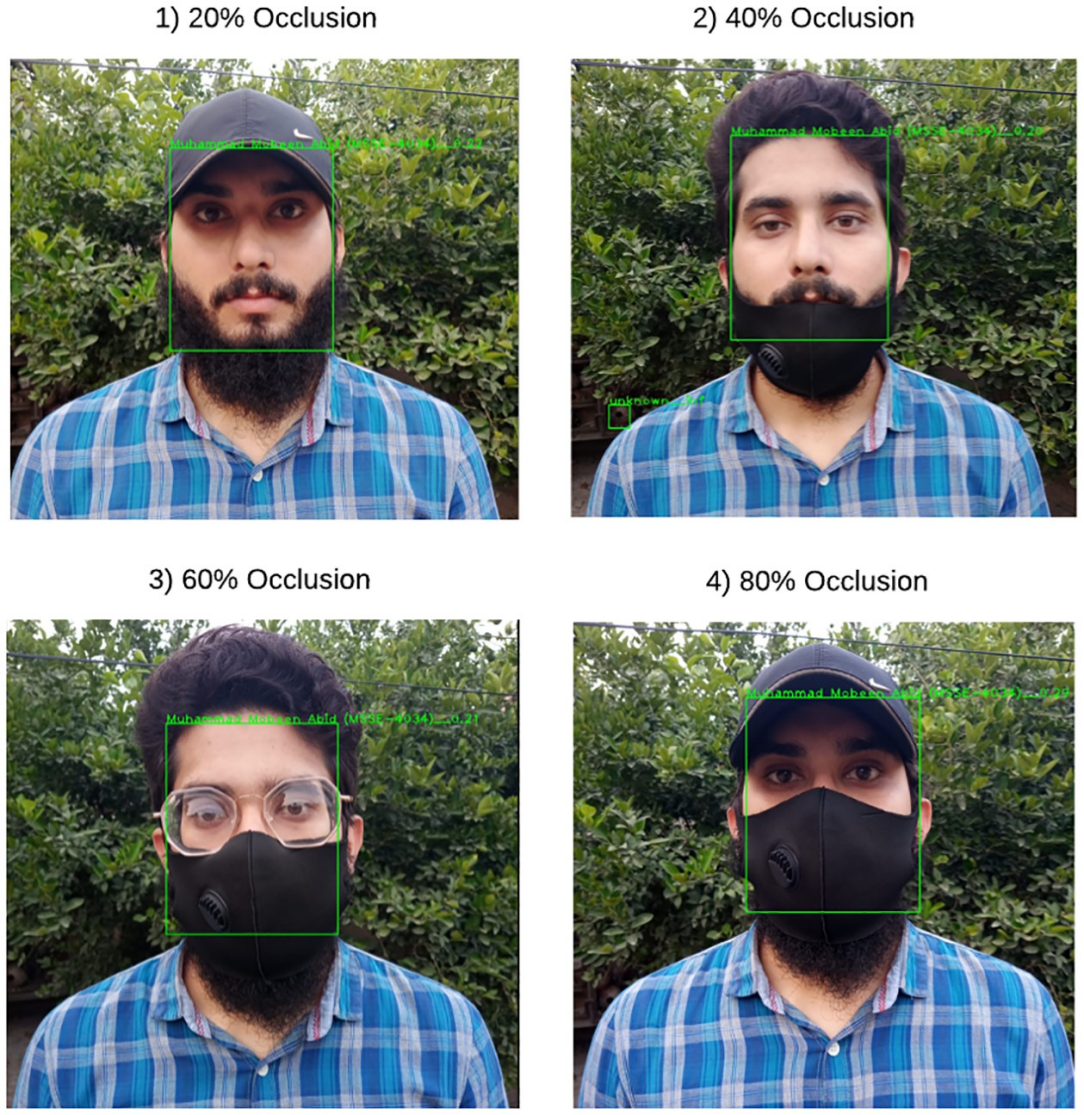
Single face recognition on different occlusion rate.

Fig 13 represents the facial recognition results using RetinaFace fused with FaceNet of a single person’s face in various poses (such as straight, side pose, face up, face down). Furthermore, the experimental result correctly shows that the recognition constraints of a human face. If the facial parameter is smaller than the threshold obtained by model training, the algorithm determines that the identifying facial feature can be accurately recognized. If the facial parameter is higher than the threshold acquired by model training, the algorithm does not recognize the corresponding face. Fig 14 displays the facial recognition outcomes of the proposed method in this research when the rate of face obscurity differs (such as 20%, 40%, 60%, or 80%).

**Fig 15:**
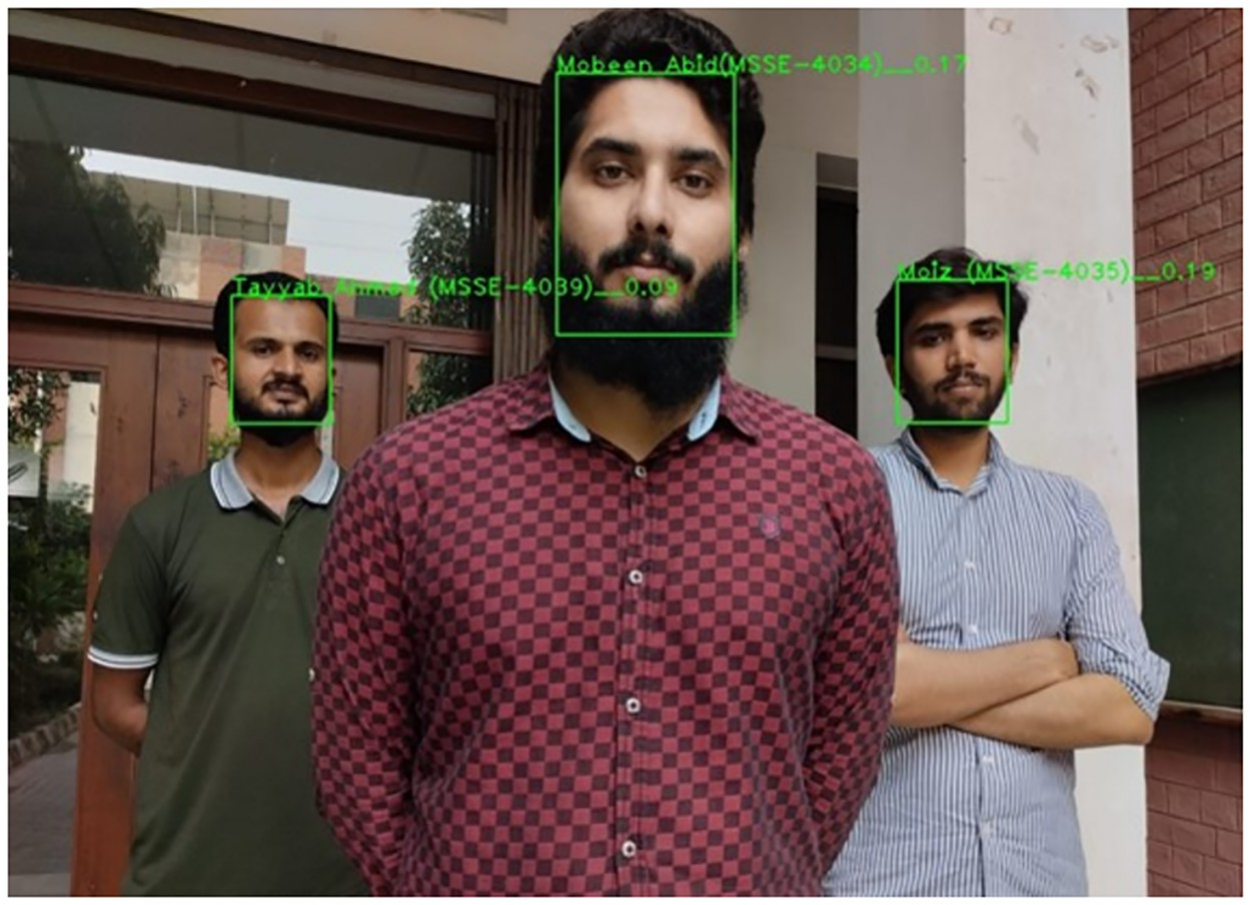
Recognize multiple human faces.

Fig 15 displays the detection and identification outcomes of the method for several human faces to demonstrate the method’s applicability in a multi-face situation. Fig 15 illustrates how the system can successfully identify even lower-resolution faces and many faces. Additionally, the model does fine regression on the faces it has identified. This research compares several well-known deep learning-based facial recognition methods. Table 3 shows the comparison outcomes. It is clear that the recognition speed of RetinaFace fused with FaceNet achieves the highest accuracy in a real-time environment, and the hybrid technique’s accuracy is better than other deep learning models; furthermore, the proposed method needs to verify its performance and efficacy.

**Table 3:** Performance comparison between face recognition techniques.

Method	Test samples	Test time, sec	Recognition accuracy, %
VggFace	2650	57.43	97.27
FaceNet	2650	54.27	97.83
MTCNN + FaceNet	2650	47.94	99.80
RetinaFace + Facenet	2650	35.39	99.86

After the experiments with the proposed method and state-of-the-art methods on the proposed dataset, it can be concluded that RetinaFace fused with FaceNet performs well in every condition and achieves high accuracy. Table 4 shows the comparison outcomes between baseline models of deep learning and the proposed method. It is clear that the recognition speed of RetinaFace fused with FaceNet achieves the highest accuracy in a real-time environment, and the hybrid technique’s accuracy is better than that of other deep learning models.

**Table 4:** Performance comparison between baseline models of deep learning and the proposed method.

Method	Test samples	Test time, sec	Recognition accuracy, %
[51]	1260	39.43	95.30
[52]	2110	36.20	92.25
[53]	1328	37.94	99.85
Proposed Method	2650	35.39	99.86

## 5. Conclusions

Real-time security surveillance in the education sector has two crucial components: facial detection and facial recognition. To address the challenges of traditional techniques for extracting facial features containing complex backgrounds, this research presents a RetinaFace-based facial detection method for locating faces in digital images. Additionally, the detected face is verified through FaceNet. FaceNet in combination with RetinaFace provided quick detection and recognition of faces having different poses and occlusion rates. The experimental findings demonstrate that the enhanced FaceNet has a 1~4% greater recognition accuracy compared to existing facial recognition techniques. In addition, the solution proposed in the current study provides more speedy recognition than other techniques to fulfill the requirements of real-time detection. Consequently, the proposed solution has a high level of application in real-time security surveillance and can play a significant role in improving the performance of real-time security surveillance.
